# Therapeutic Researches on the Action of Cod Liver Oil, and the Iodide and Bromide of Potassium

**Published:** 1855-09

**Authors:** 

**Affiliations:** Union Medicale


Therapeutic Researches on the Action of Cod Liver Oil, and the Iodide and Bromide of Potassium. Being an extract from a memoir read before the Imperial Academy of Medicine. By Dr. Lunier, (Union Meditale.) Translated, with remarks by J. J. Twheatt, M. D., Petersburgh, Va.

I do not intend, in this paper, to enter into an examination of all the diseases in which the cod liver oil, and iodide and bromide of potassium are employed; I shall only speak of the modus operandi of these therapeutic agents in those affections in which their action is most conspicuous, and at the same time, will expose the results of my own observations in those diseases in which these remedies have not heretofore been used. I shall examine successively:

1. Emaciation, constitutional and symptomatic.
2. Diseases of the Pancreas.
3. Obesity.
4. Phthisis.
5. Chlorosis.
6. Syphilitic and scrofulous affections.
7. Goitre and glandular engorgements, tumors.
8. Rickets.
9. Diabetes.
10. Coryza.
11. Amenorrhœa, dysmenorrhœa.
12. Diseases of the fœtus, children at the breast.

1. *Emaciation, Constitutional and Symptomatic.*—It is now well known that emaciation is not incompatible with the enjoyment of health, and therefore uncomplicated with any organic lesion; this condition is, however frequently symptomatic of some acute or chronic malady. The first condition I shall denominate constitutional, and may be the result of various causes, viz: 1st, absence and insufficiency of nutriment; 2nd, an extravagant expenditure of the nutritious elements, which already exist. In the first class may be included forced or voluntary abstinence; aliment of bad quality, and in some cases, the abuse of tobacco. I have stated in the first part of this memoir how, in cases of this nature, emaciation ensues; the oxygen not finding a sufficient quantity of combustible aliment in the blood, consumes the hydro-carbonaceous matters accumulated in the tissues, and emaciation is the necessary consequence.

With respect to emaciation which follows from an immoderate use of tobacco, it is only observed in persons in whom the salivary secretion is abundant; for it is to this secretion that we must ascribe the digestion of all amylaceous substances destined to serve as food for pulmonary combustion. Among the causes of the second class, I will mention great heat, laborious application, incessant fatigue, masturbation, spermorrhœa, deprivation of sleep, progress of age. Here, it is not to the absence of food that emaciation is attributed; but to the increased activity of the functions of the economy.

Incessant application and constant fatigue require efforts which augment the quantity of oxygen absorbed by the lungs, and we have shown that the more oxygen absorbed by the lungs, the larger quantity of fatty matter is consumed by the economy, and consequently emaciation follows with great facility. Emissions, of whatever nature they may be, necessitate a process of reparation, in order to supply the waste which the organism has sustained; and that process of reparation is nothing more than combustion and oxydation of the fatty matter deposited in the cellular tissue; adding to this, the influence of the nervous system, which being deranged by emotions, and all kind of perturbations, cannot supply the digestive organs with that necessary energy for the normal play of their functions. May we not often ascribe this nervous debility, this deperdition of nervous influence, to the effects of prolonged fatigue, strong emotions, constant intellectual labor, and many other causes of the same character? If it were sufficient, in the cases I have enumerated, to suppress the cause of the disease, the task of the physician could be easily accomplished; but often this will not suffice to restore the equilibrium of the organic functions that have suffered the most. And here we find indications for the use of cod liver oil, or the mixture of bromide and iodide of potassium, associated with an analeptic regimen. The iodide and bromide of potassium by their action favor the transformation of the food into combustibles, and especially fatty matter.

There are few physicians in large cities who are not often consulted for cases of the above description. Ladies often demand some remedy to arrest the emaciation which is destroying their beauty and grace; it is in these cases that the chocolate of the bromide and iodide of potassium acts in a happy manner.

But, it must be remarked, that their action is slow, that their effects are scarcely perceived before the end of the first month, and it should be continued for two or three weeks after this period. We have observed that emaciation is often symtomatic of certain morbid affections, and especially of chronic diseases. Generally, in these cases, the treatment ought to be directed against the organic lesions, of which emaciation is only a manifestation; yet when the lesion is only suspected, the treatment of symptoms is only practicable, and hence, the bromo-iodine medication is applicable; but it is chiefly during convalescence that emaciation demands an especial treatment, as the following case will show.

During the year 1851, I was consulted in the case of a young girl, aged 7, who after an attack of variola and diphtheritis, was reduced to a state of great debility, and extreme emaciation which for a long time caused much uneasiness to her parents; a minute examination failed to detect any organic derangement, and yet she emaciated daily, she could with difficulty walk, and large eschars formed on the sacrum, which was an additional cause of weakness; on the 8th of January, I prescribed the iodide and bromide mixture, in a table-spoonful of olive oil every morning; at the end of six weeks the patient was able to walk without much difficulty, and at the end of two months the case was cured.

2. *Diseases of the Pancreas.*—Among organic affections of which emaciation is of necessity a symptom, there are none in which it is more evident than in diseases of the pancreas. This peculiarity has been noticed by all those physicians who have made the diseases of this viscus a subject of study, and up to the present epoch this phenomenon had remained inexplicable. It is to the brilliant discoveries of M. Cl. Bernard that we are enabled to explain this apparent anomaly, and also other symptoms of the diseases of the pancreas. In the commencement of the diseases of this organ, there is rather an augmentation than diminution of the pancreatic secretion; it is, therefore, not at this period that emaciation is observed, but when the disease is chornic, and the organ does not perform its functions; in this condition the fatty substances become foreign bodies, and are expelled with the fœcal matters, in which they can be easily seen. Diarrhœa and constipation so common in the affections of the pancreas, can be explained by the discovery of Cl. Bernard. It is unnecessary for me to say that in such cases all fatty matter must be prohibited. The iodide and bromide preparations are here applicable; they can in some cases rouse the pancreas to action, but they should be used with caution.

3. *Obesity.*—I have already spoken in the first part of this memoir, of the causes, mode, and accumulation of fat in the cellular tissue, and the parenchymatous structure of organs. When the accumulation of fat becomes excessive, it is to be as much dreaded as the opposite condition, viz: extreme emaciation. A remedy to remove this condition is much to be desired, and up to this period, the means which have been employed have not produced the desired effects, and the economy has often suffered from the ineffectual attempts. If it were possible to act directly on the pancreas, and lessen the activity of its functions without any serious results, the object could be easily obtained; but unfortunately this cannot be; experience has taught me that in this respect opium has a diametrically opposite effect to the bromo iodide preparations, and I believe that this agent diminishes the activity of the pancreatic secretion more than any other.

Those physicians who have employed the iodide of potassium in large doses, have known emaciation to follow. The pancreatic secretion is not the only one where this peculiarity is observed; on keeping up an energetic and prolonged action, this function may not only be diminished, but completely suppressed; hence it is not surprising that Drs. Graefe and Bety should have suggested the idea of using iodine to combat polysarcia.

4. *Phthisis Pulmonalis.*—It is in phthisis that the cod liver oil has been used with the most success; but as this success has been called into question by some respectable practioners, I thought it worthy of interest to enter into an investigation and see if we cannot explain this apparent contradiction. I have already shown that the cod liver oil acts only by the fat and alkaline salts which facilitate its absorption and excite the pancreatic secretion; introduced in this way into the system, the fat serves as food to the lungs. This is I believe the whole secret of its action in consumption; and is the cause of its success and failure. The fat thus introduced into the system, arrests the progressive and rapid emaciation which accompanies this fatal disease. It must be also recollected, that combustion takes place at the same time in the lungs and general capillary system. Hence, it is easy to understand that when this process of combustion is rendered slower, in consequence of lesions of the lungs, it is more active in the capillary system, where is also produced a great quantity of carbonic acid and serosity, hence we find diarrhœa, and infiltration of the organs and cellular tissue, so often attendant in phthisis. From this slowness of combustion in the cells of the lungs, the arterial blood should contain a larger quantity of fatty matter; and may not the fatty condition of the livers of consumptive patients be ascribed to this condition of the blood?

We will now notice the failures which have alarmed some physicians; and we would ask, is it not one of the first principles of the art of cure to prescribe, in all acute affections, repose to the affected organ? The cod liver oil by its fatty matter increases the functions of the lungs; it will, therefore, be contra-indicated in phthisis, where there is acute action, or where the disease is disposed to run a rapid course.

It is not then *exclusively* as some physicians pretend that in such and such periods of consumption the cod liver oil is indicated; its action will be more effectual, more certain, when the disease progresses slowly, and free from fever. Observing this rule I prescribe this remedy less frequently than many practitioners, and I have rarely met with those accidents which doubtless resulted from an improper administration of this agent.

Permit me to say something about these agents which have been used in lieu of the cod liver oil. M. Marchel in 1844, and at a later period, M. Personne in 1850, attributed the therapeutic effects of cod liver oil to the iodine which it contains, and prepared to use in lieu of it, an oil in which the proportion of iodine should always be the same.

Mr. Marshel's preparation is as follows:

℞. Iodine, grs. $18\frac{43}{100}$;

Oil Sweet Almonds, Oct. j.

A gramme (about a half teaspoonful) of this mixture and a sufficient quantity of water to make an emulsion, to be used in place of the cod liver oil.

Mr. Marshel in recommending this oil, had only in view the introduction into the system of iodine, the oil being only a recipient. Though based on the same principle, the iodic oil of M. Personne fulfills two purposes, viz: the introduction of iodine, and also of fat into the system, and is given in the same doses. With regard to the iodide of starch of M. Quesneville, without entering into a discussion of its value, we will say that it cannot be substituted for the cod liver oil; moreover, (as has been remarked by MM. Derroult and Mialhe,) the use of this remedy may produce bad results. The iodine being set free by the conversion of the starch into sugar in the stomach, irritates the digestive organs before forming the alkaline iodide. The iodine and bromine of the cod liver oil have not only for effect to facilitate the digestion of fat, they impress upon all the organic functions an increased activity of function, which ultimately tends to aid the action of the hydro-carbonaceous matters; but, the cod liver oil is not the only agent containing iodine, which has been employed in consumption; it has been detected in Iceland moss, etc., and I have no doubt that the remarkable results which have been obtained by Amedie Latour in the use of common salt, in the treatment of phthisis, are owing to the iodine and bromine which it contains. Is it not in this manner we must explain the action of common salt in the fattening of cattle? and if success has not invariably resulted, we should attribute it to a deficiency of iodine and bromine. What has been said with regard to the action of sea salt, applies equally to the mineral waters; they have an action on the animal economy peculiarly their own, stimulating and regulating the organic functions, restoring vigor, reestablishing the secretions, and eliminating from the system foreign matters. I am persuaded that most of these waters owe in part, if not the whole of their efficacy, to the iodine and bromine which enter into their composition. Whatever formula you may employ in administering cod liver oil and its kindred preparations, I do not believe that the iodine acts directly on the pulmonary lesion; it assists nature, it rouses the organism to activity, which facilitates the cretaceous transformations of the tubercle, and the cicatrizations of caverns; in such cases to furnish to the economy the elements which are deficient, to prolong life, is accomplishing a great deal; but, it is doing more when it enables nature to finish the work of elimination and repair.

For fifteen years many distinguished practioners, particularly MM. Andral, Bricheteau, Depasquier, have employed the iodide of iron with success in phthisis. I have myself in some cases where chloro-anemic symptoms predominated, used the iodide of iron with favorable results. I think that the iodide neutralizes the sometimes prejudicial effects of the iron.

5. *Chlorosis.*—It is not alone in consumption with chloro-anemic symptoms, that the bromide and iodide of iron are indicated; there are certain forms of chlorosis accompanied with puffiness of the face, anorexia, emaciation, dysmenorrhœa, in which they ought to be preferred above all other ferruginous preparations. In cases of this description, even when other means have failed, this medication will restore the functions to their normal condition; a few weeks are sufficient to restore the appetite, destroy the pallor, increase the forces, and remove all the symptoms of chlorosis.

6. *Syphilis, Scrofula.*—Since the interesting labors of Drs. Wallace and Ricord, the iodide of potassium has been so commonly used in the tertiary forms of syphilis, that it will be useless for us to say anything on this therapeutic point; the bromide of potassium has likewise been used with success.

I can affirm that during my services at the hospital (Du Midi), that its action was far from being as prompt and certain as the iodide; we should not, however, abandon it. My experience warrants the assertion that more satisfactory results are obtained by a combination of the two remedies. With a few grains of the iodide and bromide, greater effects will be produced than four or five times the same quantity administered separately. The scrofulous affections bear too strong an analogy to the tertiary forms of syphilis for the preceding remarks not to be applicable. But what is the mode of action of the iodide of potassium in syphilis? Does it act as M. Dorvault pretends, by its fluidifying and antiplastic properties? I would rather attribute its therapeutic effects to the increased activity of the capillary circulation and the secretions, which disembarrass the organism of the morbific elements that it contains, and at the same time the digestive functions are so modified as to be able to restore to the system what it has lost by the skin, the kidneys, and pulmonary surfaces. In certain cases of scrofula the bromo-iodides are not sufficient, and we are obliged to resort to cod liver oil, which I am in the habit of making more active by adding 5 parts of the iodide and bromide of potassium to 100 parts of cod liver oil. It should never be forgotten that cod liver oil is absorbed by the surfaces of old ulcers, blisters and the skin deprived of its epidermis, and that it acts almost as efficaciously as when administered in the usual manner. I suspected that it was thus introduced into the system by witnessing the beneficial effects of dressing old ulcers with the oil—a treatment which I strongly recommend in all ulcers of a rebellious character, particularly those situated in the sacrum and trochanters. In patients long confined to bed, we should likewise attribute to the absorption of the oil the happy results obtained by M. David in the treatment of the diseases of the skin of a chronic character.

7. *Bronchocele, Glandular Engorgements.*—I believe that in the treatment of goitrous engorgements and tumors of various characters, the iodide and bromide of potassium act by increasing the activity of the secretions and capillary circulation. In engorgements of the cervical glands, I have found that the addition of iron to the bromo-iodine mixture acted well even when there were no symptoms of chlorosis.

8. *Rickets.*—After consumption rickets appears to be the disease in which cod liver oil has been more used and with more success. At the same time that Schenk and Fehr were experimenting in Germany, MM. Bretonneau, Bonnett, Guersant, and Trousseau, were obtaining wonderful results in France. The remedy now became popular, and there are now few practitioners who have not employed it in children as well as in adults. At first the results were ascribed to the phosphate of lime (phosphore) which the cod liver oil contained, but it is now well ascertained that cod liver oil rarely contains this agent. Moreover, MM. Bretonneau, Dubois, and Pophen have demonstrated that similar results can be obtained by other oils. It is not therefore to the action of iodine, or bromide, or phosphate of lime, that these effects are to be attributed, but simply to the fat of the oil. In order to facilitate the absorption of fat, it is well to associate the iodide and bromide of potassium. At first it is difficult to comprehend how the introduction of fat into the animal economy can modify so rapidly the nutrition of bones, and restore the functions to their normal standard. I believe with Soemmering, Heyne, and Heckeren, that rickets arises from a want of equilibrium between the elements deposited in the tissues and those taken up by the absorbents; the fat restores the equilibrium.

9. *Diabetes.*—MM. Combette and Martin Solen have employed, the first, the iodide of iron, the second, the iodide of potassium, in the treatment of diabetes. The mode of action of these medicines ought to have, it seems to me, destroyed all expectation of success; these preparations have for a constant effect increased action of the salivary glands; and is not the saliva the solvent of amylaceous substances? If, therefore, there be any therapeutic agent diametrically the opposite in its action to that of iodine, that agent should be preferred in the management of diabetes; that agent, for me, is opium. And here the theory is in accordance with facts, for the preparations of opium have been used thus far with more success than any other medicines.

10. *Coryza, Ozena.*—The bromo-iodine medication occasions almost constantly an irritation of the nasal mucous membrane, a hypersecretion and frontal cephalalgia, in other words, a commencement of inflammation; it is not, therefore, empirically that we have used these preparations in the treatment of the above diseases; cauterization of the ulcer by the nitrate of silver, or the acid, assists greatly the action of these remedies.

11. *Amenorrhœa, Dysmenorrhœa.*—The preparations of iodine and bromine having the effect of augmenting the activity of the digestive functions and capillary circulations, and producing a uniform distribution of the nutritious elements of the blood throughout the animal economy, are naturally indicated in a majority of cases of amenorrhœa and dysmenorrhœa; they produce a healthy modification of the general system, and a determination of blood to the uterine organs; a few grains of aloes, and a few leeches to the internal surfaces of the labia majora will be good adjuvants. Since I have adopted this method of treatment I have found few refractory cases; and often obtained a prompt cure, when other methods of treatment have failed. My treatment is as follows: Every morning I prescribe for one or two months, of the following mixture:

℞. Iodide of potassium; Bromide of potassium, ana, gr. v.; Artemisia vulgaris; Distil. water of mint; or the antimisia, ana, ℥ss.; Fiat solut.

At certain periods, according to individual peculiarities, I prescribe nine pills of aloes, containing one grain in each pill, all to be taken in three days. On the fourth day I apply two to four leeches to the sacro-lumbar region. If there be any symptoms of anemia, I add iron to the prescription; in addition to its general effects, I believe that these medicines have a specific influence on the uterine functions.

12. *Pregnancy, Nursing.*—M. Coindet in his first memoir on the use of iodine, advises that it should not be employed in pregnant women, because it might determine hemorrhage. In spite of this wise recommendation, M. Delprayse thought proper to use it in malformation of the pelvis, with the view of retarding the development of the fœtus: in the cases reported by Dr. Delprayse in support of this opinion, the women were affected with rickets, for which iodine had been administered during the two last months of pregnancy. Should we not rather ascribe the happy results to an amelioration of the general health, rather than to the action of iodine? I beg leave to call attention to the fact that iodine and bromine, like all mineral substances, are found in all of the secretions, the milk as well as the saliva and urine;—these medicines, when administered to the mother, will act on the infant. I will not insist on the importance of this physiological fact and the indications which it furnishes. Many times have I prescribed for infants, and adults, too, the milk of the goat and jennet; these animals being fed on food containing the bromo-iodine salts.—*Virginia Med. and Sur. Jour.*

